# Peracetic Acid Treatment Generates Potent Inactivated Oral Vaccines from a Broad Range of Culturable Bacterial Species

**DOI:** 10.3389/fimmu.2016.00034

**Published:** 2016-02-11

**Authors:** Kathrin Moor, Sandra Y. Wotzka, Albulena Toska, Médéric Diard, Siegfried Hapfelmeier, Emma Slack

**Affiliations:** ^1^Institute for Microbiology, ETH Zürich, Zürich, Switzerland; ^2^Institute for Infectious Disease, University of Bern, Bern, Switzerland

**Keywords:** oral vaccines, inactivated vaccines, *Salmonella typhimurium*, *Yersina enterocolytica*, IgA

## Abstract

Our mucosal surfaces are the main sites of non-vector-borne pathogen entry, as well as the main interface with our commensal microbiota. We are still only beginning to understand how mucosal adaptive immunity interacts with commensal and pathogenic microbes to influence factors such as infectivity, phenotypic diversity, and within-host evolution. This is in part due to difficulties in generating specific mucosal adaptive immune responses without disrupting the mucosal microbial ecosystem itself. Here, we present a very simple tool to generate inactivated mucosal vaccines from a broad range of culturable bacteria. Oral gavage of 10^10^ peracetic acid-inactivated bacteria induces high-titer-specific intestinal IgA in the absence of any measurable inflammation or species invasion. As a proof of principle, we demonstrate that this technique is sufficient to provide fully protective immunity in the murine model of invasive non-typhoidal *Salmonellosis*, even in the face of severe innate immune deficiency.

## Introduction

Many immune mechanisms controlling bacterial infection in the blood and systemic secondary lymphoid organs are well described and understood (1, 2). As the systemic immune system is exquisitely sensitive to bacterial-derived “pathogen-associated molecular patterns” (PAMPs) and antigens, parenteral introduction of very low concentrations of live or inactivated bacteria induces high titer serum IgG responses and T cell activation in experimental animals (3). These simple vaccination protocols have permitted the elucidation of major effector functions, including antibody-mediated enhancement of phagocytosis and pathogen recognition (2), T cell help to orchestrate appropriate cell-mediated antimicrobial responses (4, 5), and enhancement of the microbicidal activity of the complement system (1). Correspondingly, we have been able to identify a large number of mechanisms by which bacterial pathogens subvert and evade systemic immunity and can start to apply this knowledge clinically (6).

By contrast, our knowledge of immune effector function at mucosal surfaces, particularly in the intestinal system, remains incomplete (7, 8). The mammalian large intestine is home to an extraordinarily dense microbial consortium, known as the microbiota. Among other functions, these microorganisms determine the host nutrient profile (9), densely occupy intestinal niches providing “colonization resistance” to infection (10), and determine the immune status of the host (11). Coevolution has produced an intestinal immune system that is both compartmentalized away from the systemic system such that parenteral immunization does not induce robust mucosal immunity (12) and rather insensitive to locally delivered bacterial PAMPs and antigens in order to coexist with the commensal microbiota (11, 13). In comparison to activation of systemic immunity, it is therefore a considerable challenge to activate the intestinal immune system, in particular to induce high-affinity secretory IgA, the major antibody isotype secreted into the intestinal lumen.

In order to induce strong intestinal immunity, it is necessary to deliver both antigen and appropriate adjuvant-derived signals into the gut-associated lymphoid tissues [recently reviewed in Ref. (14)]. The most potent strategies to induce bacteria-specific IgA employ live-attenuated invasive pathogens [e.g., S*almonella enterica* ssp. *enterica* ser. Typhimurium (15), *Shigella* species (16)]. This had led to the suggestion that some degree of pathogenicity, such as the ability to invade into the epithelium or subvert phagocyte function, is required for induction of intestinal immunity (17–21). While mucosal adaptive immunity can easily be activated by these vaccines, the vaccination process itself is often associated with mild inflammation, persistent colonization of secondary lymphoid tissues, and shifts in microbiota composition, due either to live vaccine presence or inflammatory processes (18, 22, 23). Any major perturbation of the microbiota has the potential to modify a very broad spectrum of host physiological functions (11), necessarily complicating the dissection of effector mechanisms. Furthermore, it is difficult and generally undesirable to generate an invasive pathogen from an apathogenic species in order to study immunity in host–commensal interactions.

An alternative strategy employed by us and others, particularly in the study of commensal microbes, is the gavage of high numbers of live apathogenic bacteria (24–27). For example, specific IgA is induced by six oral doses of 10^10^ live *Escherichia coli* K-12, which is a lab-adapted strain free of any identifiable virulence mechanisms (13). This effect can be mimicked by monocolonizing germ-free mice with very low numbers of apathogenic bacteria, providing those bacteria grow up to a high density in the otherwise uncolonized intestinal lumen (13, 25). Therefore, pathogenicity is not absolutely necessary for induction of specific IgA responses if high loads of live bacteria are present. Again, the drawback of this method for IgA induction is that the animal tends to be permanently colonized with the vaccination strain from the initiation of vaccination (24–27). This makes it very difficult to clearly dissect effects on phenotype, colonization levels, etc. due to niche occupancy and shifts in host physiology. Elegant work with auxotrophic mutations can generate systems where the vaccination strain does not permanently colonize a germ-free mouse, permitting later re-challenge and study (13, 28). However, this requires powerful genetic systems in your organism of choice as well as a very detailed knowledge of bacterial metabolism and potential escape mechanisms.

The use of fully inactivated oral vaccines is, therefore, highly attractive as there is potential to induce a specific mucosal immune response without inducing inflammation and without persistently colonizing the intestine and/or associated lymphoid tissues. In the murine system, inactivated oral vaccines have often been found to be ignored by the mucosal immune system (13, 27). However, inactivated oral vaccines are so far the most successful strategy to induce at least partially protective immunity against enteric bacterial pathogens in humans (29). The human cholera vaccines Shanchol^®^ and Dukoral^®^ rely on oral delivery of more than 10^10^ inactivated *Vibrio cholerae* in the presence or absence of the mucosal adjuvant recombinant cholera toxin B subunit (30). An enterotoxigenic *E. coli* vaccine currently in clinical trials (31) is also based on oral delivery of inactivated bacteria along with the heat-labile toxin, a homolog of cholera toxin with known mucosal adjuvant activity. An inactivated *Shigella flexneri* vaccine is also showing promise in humans (32). It is, therefore, clear that successful oral vaccination of humans can be achieved in the absence of live bacteria; at least if a known mucosal adjuvant is present.

It is unclear why this discrepancy exists in the dogma between human and murine oral vaccination. Nevertheless, mouse infection/colonization remains the most commonly used system to elucidate biological mechanisms of host–microbe interactions, as experiments can be carried out that are simply not possible in human patients. We hypothesized that previous failure of inactivated oral vaccines in murine systems may be due to quantitatively insufficient delivery of antigens and PAMPs. Our previous work had determined that both the dose and the particulate nature of the vaccine were important to induce specific IgA (13). Therefore, in order to produce oral vaccines at concentrations of more than 10^10^ inactivated bacteria per 100 μl dose, we needed a drastic inactivation method that nevertheless minimized bacterial lysis. To this end, we made use of the very strong oxidizing agent peracetic acid (33–35). We were able to fully inactivate a taxonomically diverse range of bacterial species to generate particulate oral vaccines. As a proof of principle, we here demonstrate induction of high-titer IgA responses against a range of Enterobacteriaceae species.

In order to compare this strategy to existing oral vaccines, we made use of the murine model of invasive non-typhoidal *Salmonellosis* (36). This is a particularly challenging model for vaccination-mediated protection as <10 CFU delivered orally results in lethal infection within 5 days (36), and live-attenuated vaccine strains are known to cause severe pathology in mice with innate immune deficiencies (37). Using our peracetic acid-inactivated *S*. Typhimurium vaccine, we could generate high-titer IgA in the absence of any detectable intestinal pathology, and could observe sterile protection from disease, even in the mouse model of chronic granulomatous disease.

## Materials and Methods

### Ethics Statement

All animal experiments were approved by the legal authorities (licenses 223/2010 and 222/2013; Kantonales Veterinäramt Zürich, Switzerland) and performed according to the legal and ethical requirements.

### Mice

SOPF C57BL/6, J_H_^−/−^ (38), *cybb*^−/−^ (39), IgA^−/−^ (40), and TCRβδ^−/−^ (41) mice (all C57BL/6 background) were re-derived by artificial insemination into a specific opportunistic pathogen-free (SOPF) foster colony to normalize the microbiota and bred in full barrier conditions in individually ventilated cages in the ETH Phenomics center (EPIC), ETH Zürich, for <4 generations. Specific pathogen-free mice (SPF) wild-type C57BL/6 mice were bred at the Rodent center HCI (RCHCI), ETH Zürich, in individually ventilated cages. Low complex microbiota (LCM) mice (C57BL/6 background) are ex-germ-free mice, which were colonized with a naturally diversified Altered Schaedler flora in 2007 (23) and were bred in individually ventilated cages under strict hygienic isolation at the RCHCI ETH Zürich. All mice were used between 6 and 12 weeks of age. Wherever possible, male and female mice were randomized between groups to permit detection of a gender-specific effect.

### Bacterial Strains and Growth Conditions

For infection experiments, the streptomycin-resistant wild-type strain *S*. *enterica* serovar Typhimurium (SL1344 wild-type clone SB300) or the isogenic *sseD::aphT* SPI2 mutant *S*. Typhimurium (M556) [described previously in Ref. (42, 43)] were cultured in LB for 12 h at 37°C and subcultured for 3 h as described previously (44). For vaccine production and antibody titering, all strains (Table 1) were cultured overnight in LB medium with aeration to late stationary phase.

**Table 1:** **Bacterial strains used in this study**.

Species name	Strain name	Genetic modifications/resistances	Source
*Salmonella enterica* serovar Typhimurium	SB300	None	Murine passage of the type-strain SL1344 (44)
		Streptomycin resistant	
*Salmonella enterica* serovar Typhimurium	M2702	Δ*sseD*::ΔinvG	SB300 derivative (50)
		Streptomycin resistant	
*Salmonella enterica* serovar Typhimurium	M556	*sseD::aphT*	SB300 derivative (50)
		Streptomycin resistant	
		Kanamycin resistant	
*Salmonella enterica* serovar Typhimurium	SKI10	*wbaP::aphT*	SB300 derivative (50)
		Streptomycin resistant	
		Kanamycin resistant	
*Salmonella enterica* serovar Enteritidis	125109	Wild type	(62)
*Salmonella enterica* serovar Choleraesuis	ATCC25957 (914/99)	Wild type	(63)
*Citrobacter rodentium*	DSM 16636	Wild type	(64)
*Yersinia enterocolitica*	JB580	Wild type	Kind gift from Prof. Markus Aebi, ETH Zurich (65)
*Klebsiella pneumonia*		Human fecal isolate	(66)
*E. coli*	Nissle 1914	Human isolate	(67)
*E. coli*	8178	Mouse commensal isolate	(23)
*Burkholderia multivorans*	AE1064722	Cystic fibrosis patient lung isolate	Kind gift of Dr. A. Endimiani, University of Bern
*Moraxella catarrhalis*	O35E	Human isolate	(68)
*Staphylococcus aureus*	NCTC 8532	Human lung isolate	ATCC
*Staphylococcus epidermidis*		Human skin isolate	(69)
*Staphylococcus xylosus*		Mouse fecal isolate	(45)
*Enterococcus faecalis*		Mouse fecal isolate	(45)
*Enterococcus faecalis*		Human fecal isolate	(69)
*Pseudomonas aeruginosa*	PA01	Wild-type strain	Institute strain collection
*Pseudomonas fluorescens*			Institute strain collection

### Testing of (a) Paraformaldehyde Fixation, (b) Mild Heat-Treatment, and (c) Hydrogen Peroxide-Mediated Vaccine Inactivation

One liter of LB was inoculated with avirulent *S*. Typhimurium and cultured overnight at 37°C with shaking. The bacteria were concentrated by centrifugation at 16,000 × *g* for 20 min and resuspended in 10 ml D-PBS without Calcium or Magnesium (e.g., 14190-094, Gibco, Waltham, MA, USA). The 500 μl aliquots were then made in 2 ml snap-cap tubes (Sarstedt, Nümbrecht, Germany). Three aliquots were subjected to the following treatments: (A) heat-treatment (1 h, 60°C with mild agitation in an Eppendorf Thermomixer); (B) PFA fixation [the bacteria were pelleted by centrifugation at 16,000 × *g* and resuspended in 1 ml of 4% paraformaldehyde (158127, Sigma-Aldrich, St. Louis, MO, USA) in D-PBS and incubated for 1 h at room temperature (RT)]; or (C) H_2_O_2_ inactivation [the bacteria were transferred to a 50 ml tube to contain the effervescence and 30% H_2_O_2_ (H-1009, Sigma-Aldrich) was added to a final concentration of 3%. The suspension was incubated for 1 h at RT]. This was compared to peracetic acid mediated inactivation on matched aliquots, as described in the paragraph below. After 1 h, the cultures, along with a control sample in PBS only at RT, were washed three times with 1 ml D-PBS to remove inactivating agents and were then resuspended in 500 μl for analysis. The 100 μl per sample were inoculated into 200 ml LB for overnight culture to determine sterility. The remaining sample was used for bright-field microscopy and FACS counting.

### Production of Peracetic Acid-Inactivated Vaccines

Bacteria for peracetic acid-inactivated vaccines were grown overnight to late stationary phase at their respective optimal growth conditions. Bacteria were harvested by centrifugation (16,000 × *g*, 15 min) and resuspended at a density of 10^9^–10^10^ per ml in sterile D-PBS. The 10 ml aliquots were transferred to sterile 50-ml Falcon tubes. Peracetic acid (433241, Sigma-Aldrich) was added to a final concentration of 0.4%. The suspension was mixed thoroughly and incubated for 1 h at RT. The bacteria were washed three times in 50 ml sterile D-PBS, meticulously removing all supernatant after each centrifugation step, and thoroughly resuspending the pellet each time to rapidly remove the peracetic acid. The final pellet was resuspended at a final concentration of 10^11^ particles per ml in sterile D-PBS (determined by OD_600_) and stored at 4°C for up to 3 weeks. As a quality control, each batch of vaccine was tested before use by inoculating 100 μl of the inactivated vaccine (one vaccine dose) into 200 ml LB and incubating over night at 37°C with aeration to ensure complete inactivation, i.e., a “negative enrichment culture.” One microliter of vaccine suspended in 100 μl D-PBS was used for bright-field microscopy. If aggregation was observed, the vaccine was aliquoted into sterile 2-ml tubes (Sarstedt) each with a single sterile 5-mm steal ball [5 mm G80 1.3541, PO8(xq + 8) × B2 beads, Berani Kugellager AG, Uster, Switzerland]. The tubes were then shaken at 25 Hz for 1 min in a Retsch Tissuelyser (Qiagen, Hilden, Germany) to disrupt aggregates.

### FACS Quantification of Intact Inactivated Bacterial Particles

In order to quantify the total intact bacterial particles, the OD_600_ of prepared vaccines or control live bacterial suspensions was measured. The suspensions were then diluted to give an approximate OD_600_ of 0.1 and the dilution factor noted. The 150 μl of this culture was then added to 150 μl of PBS containing and known concentration of Fluoresbrite Multifluorescent 1 μm Microspheres (Polysciences, Warrington, PA, USA) (in the range of 10^8^ per ml, which is roughly 50 μl of the delivered bead suspension in 10 ml PBS, as determined by dilution and hemocytometer counting). Samples were acquired on an LSRII Flow cytometer (Becton Dickenson, NJ, USA) with forward- and side-scatter parameters in logarithmic mode and both parameters thresholded on a low value to exclude electronic noise but detect beads and bacteria. Beads were identified based on a “beads only” sample, as highly fluorescent in all channels. Bacteria/vaccine were identified by the absence of fluorescence as compared to the “bead-only” sample. Ten thousand multifluorescent bead events were acquired for each sample and the “counts” of beads and bacteria extracted by analysis in FlowJo (Treestar, Ashland, OR, USA). The background in the bacterial gate acquired with the “beads-only” sample was subtracted from all bacterial counts. The concentration of bacteria was then calculated using the formula Bacterial density = Bead density*bacteria FACS counts/Beads FACS counts.

### Oral Vaccination with Peracetic Acid-Inactivated Vaccines

Mice received 10^10^ particles of the respective peracetic acid-inactivated bacteria in 100 μl of D-PBS by oral gavage once per week for 3 weeks. Except where stated in Figure 4, no antibiotics were applied during the vaccination period. Unless otherwise stated, on day 21 after the first gavage (i.e., 7 days after the final gavage), mice were used for analysis of antibody titers or infection experiments. For the data show in Figure 4C, mice received 0.8 g/kg ampicillin sodium salt (A0839, Applichem, Darmstadt, Germany) in sterile water or 1.0 g/kg gentamicin sulfate (A1104, Applichem) in sterile water by gavage 24 h prior to each dose of vaccine.

### Oral Vaccination with Live-Attenuated *Salmonella*

Mice were pretreated with 1 g/kg streptomycin sulfate (A1852, AppliChem) in sterile water by gavage. Twenty-four hours later, the mice were inoculated with 5 × 10^7^ CFU avirulent *S*. Typhimurium *sseD::aphT* (M556) by gavage [as described in Ref. (18)].

### Analysis of Specific Antibody Titers by Bacterial Flow Cytometry

Specific antibody titers were analyzed in mouse serum and intestinal washes by flow cytometry as described previously (45). Briefly, intestinal washes were collected by flushing the small intestine with 5 ml of a wash buffer containing PBS, 0.05M EDTA (A1104, Applichem), and 1 μg/ml Soybean trypsin inhibitor (T9128, Sigma-Aldrich). Intestinal washes were centrifuged at 16,000 × *g* for 30 min and aliquots of the supernatants were stored at −20°C until analysis. Blood was collected into tubes containing clotting activating gel (41.1395.005, Sarstedt) and allowed to clot at RT for 30 min before centrifugation at 16,000 × *g* for 15 min. Bacterial targets (antigen against which antibodies are to be titered) were grown to late stationary phase and gently pelleted for 2 min at 3000 × *g* in an Eppendorf minifuge. The pellet was washed with sterile-filtered FACS buffer [PBS, 1% Bovine serum albumin factor V (K41-001, GE Healthcare, Little Chalfront, UK), 0.05% sodium azide (71289, Sigma-Aldrich)] before resuspending at a density of approximately 10^7^ bacteria per ml. Intestinal washes and serum were heat-inactivated for 30 min at 56°C and centrifuged again at 16,000 *g* for 10 min to remove bacterial sized particles that may have been generated by the heat-treatment. Supernatants were used to perform serial dilutions. The 25 μl of the dilutions were incubated with 25 μl bacterial suspension at 4°C for 1 h. Bacteria were washed twice with 200 μl FACS buffer before resuspending in the appropriate antibody cocktail: monoclonal FITC-anti-mouse IgA (10 μg/ml, 559354, Clone C10-3, BD Pharmingen, New Jersey, NY, USA), FITC-anti-mouse IgG2b (5 μg/ml, 406706, Clone RMG2b-1, BioLegend, San Diego, CA, USA), PE-anti-mouse IgG1 (5 μg/ml, 406608, Clone RMG1-1, BioLegend), and APC/Cy7-anti-mouse IgM (5 μg/ml, 406516, Clone RMM-1, BioLegend). After 1 h of incubation, bacteria were washed once with FACS buffer and resuspended in 300 μl FACS buffer for acquisition on FACS LSRII using FSC and SSC parameters in logarithmic mode. Data were analyzed using FlowJo (Treestar, Ashland, OR, USA). After gating on bacterial particles and compensation for bleed-through between fluorescence detectors where appropriate, median fluorescence intensities (MFI) were plotted against antibody concentrations for each sample and 4-parameter logistic curves fitted using Prism (Graphpad, La Jolla, CA, USA). Titers were calculated from these curves as the inverse of the antibody concentration giving an above-background signal (see Figure S1 in Supplementary Material).

### Challenge of Vaccinated Animals with Wild-Type *S*. Typhimurium

*Salmonella* infections were performed in individually ventilated cages at the RCHCI, Zurich as described previously (44). Mice were pretreated with 1 g/kg streptomycin sulfate (Applichem) in sterile PBS by gavage. Twenty-four hours later, the mice were inoculated with 5 × 10^5^ CFU *S*. Typhimurium SB300 by gavage. As mentioned earlier, 24-h post infection, blood and intestinal lavages were collected for analysis of specific antibody titers. Bacterial loads (CFU) in fresh fecal pellets, mesenteric lymph nodes (mLN), spleen, and cecal content (CC) were determined by plating on MacConkey agar plates containing 50 μg/ml streptomycin sulfate (Applichem).

### Histopathological Evaluation

Samples of cecal tissue were embedded in OCT (Sakura, Torrqance, CA, USA) and snap frozen in liquid nitrogen. Five-micrometer cross-sectional tissue sections were cut and stained with haemotoxylin and eosin [as described in Ref. (44)]. Tissue sections were scored for cecal pathology as described (44). Briefly, the cecum pathology score is based on edema, polymorphonuclear cell infiltration, reduced numbers of goblet cells, and epithelium disruption with a maximum score of 14.

### ELISAs

Lipocalin 2 was detected in feces homogenized in 500 μl sterile PBS by ELISA using the DuoSet Lipocalin ELISA kit (DY1857, R&D Systems, Minneapolis, MN, USA) according to the manufacturer’s instructions. Total concentrations of antibody isotypes in mouse serum or intestinal lavages were determined by sandwich ELISA. Coating antibodies were goat anti-mouse IgA (1040-01, SouthernBiotec, Birmingham, AL, USA), goat-anti-mouse IgG2b (1090-01, SouthernBiotec), goat-anti-mouse IgG1 (1070-01, SouthernBiotec), and goat-anti-mouse IgM (1020-01, SouthernBiotec). Detection antibodies were HRP-conjugated anti-mouse IgA α chain (A4789, Sigma-Aldrich), anti-mouse IgG γ chain (A3673, Sigma-Aldrich), and anti-mouse IgM μ chain (A8786, Sigma-Aldrich). Standards were purified mouse IgA (03101D, Pharmingen) or mouse reference serum (RS10-101, Bethyl, Montgomery, TX, USA).

### Statistics

As we were predominantly working with datasets with *N* < 10, non-parametric tests were employed wherever possible. Where two groups of data are compared, analysis was carried out using two-tailed Mann–Whitney *U* tests with a significance cut-off of 5%. Where more than two groups were compared, data were analyzed by Kruskal–Wallis test with Dunns post-test to account for multiple testing. Where more than one variable was tested simultaneously, log-normally distributed data were normalized and tested by two-way ANOVA with Bonferroni post-tests. All statistics were evaluated using Graphpad Prism. The dendrogram was generated by multiple alignment of 16S rDNA sequences using ClustalX and NJPlot (Conway Institute UCD Dublin, Ireland). As very large effects with minimal variation were measured, power calculations were not necessary to determine sample size and we adhered to standard practice of analyzing at least five mice per group, wherever possible.

## Results

**Figure 1:**
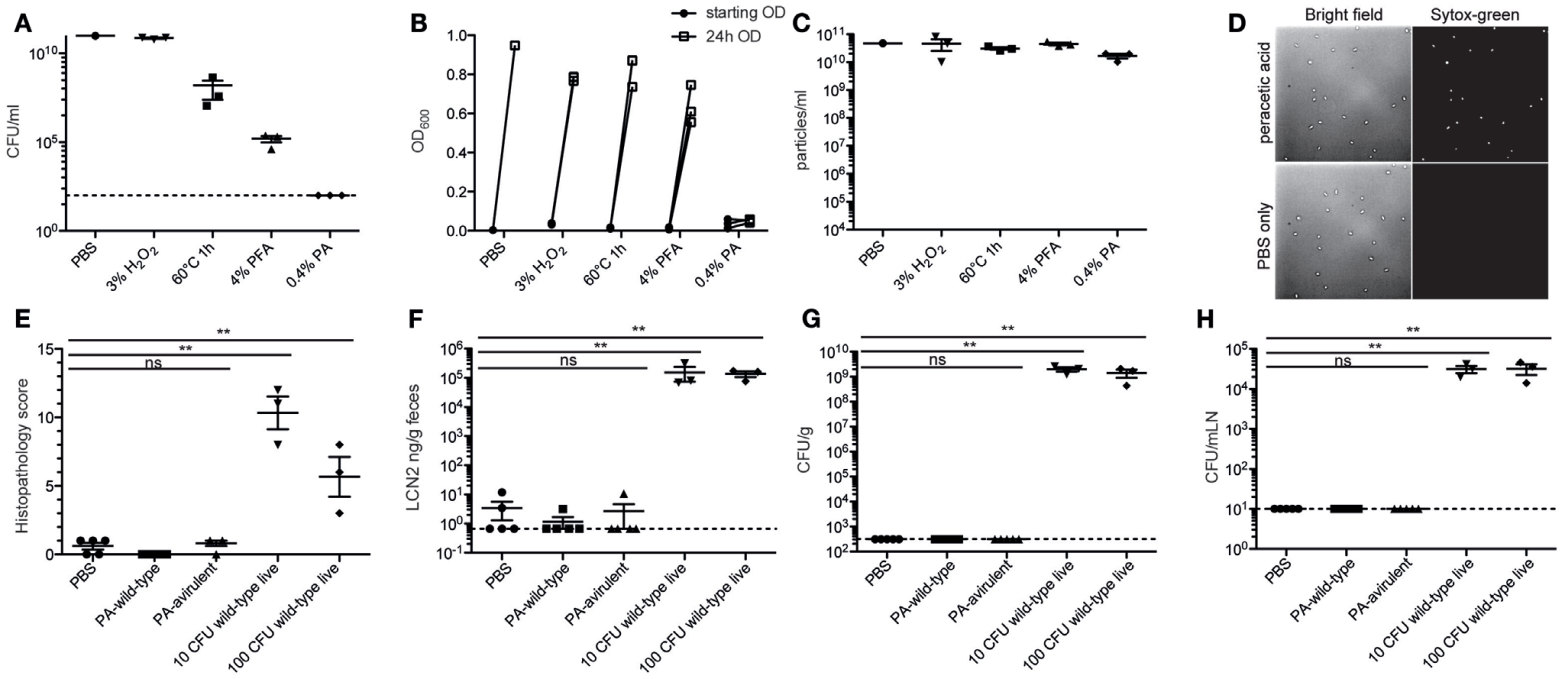
**Peracetic acid highly efficiently kills bacteria, with minimal lysis**. **(A–D)** Avirulent *S*. Typhimurium was cultured overnight to stationary phase, concentrated, and inactivated by incubating in 3% H_2_O_2_ for 1 h, heating to 60°C for 1 h, incubating in 4% PFA/PBS for 1 h, or incubating in 0.4% peracetic acid for 1 h. All aliquots were subsequently washed three times to remove fixatives/oxidizing agents before resuspending for CFU analysis by plating **(A)** and total sterility by enrichment culture **(B)**. Lysis was determined by counting bacterial particles by flow cytometry **(C)**. To examine morphology, the bacteria were diluted in PBS containing 0.5 μM Sytox-green and were imaged by bright-field and fluorescence microscopy at 100× magnification **(D)**. One representative experiment of two. **(E–H)** C57BL/6 LCM mice received 1.0 g/kg streptomycin p.o. 24 h before oral gavage of 5 × 10^10^ particles of peracetic acid-inactivated wild-type *S*. Typhimurium SB300 (PA-wild-type), 5 × 10^10^ particles of peracetic acid-inactivated avirulent *S*. Typhimurium M2702 (PA-avirulent), or 10 CFU or 100 CFU of wild-type live *S*. Typhimurium SB300. After 72 h, all animals were analyzed. **(E,F)** Intestinal pathology as determined by histopathology or fecal Lipocalin 2 (LCN2). Kruskal–Wallis test, *P* = 0.0023 **(G,H)**. Cecal content (Kruskal–Wallis test, *P* = 0.0007) and mesenteric lymph node CFU (Kruskal–Wallis test, *P* = 0.0007). **Dunn’s post-test, *P* < 0.01. One experiment with three to five mice per group.

In order to deliver 10^10^ inactivated bacteria orally with a minimal risk of accidental infection, it was essential to have a method that very efficiently kills bacteria at high densities without destroying antigenic structures. To this end, we tested several different standard inactivation procedures, including paraformaldehyde fixation (32), pasteurization (30), and hydrogen peroxide treatment (46), as well as a novel peracetic acid treatment, on the broad host-range pathogen *S. enterica* serovar Typhimurium (*S*. Typhimurium). As with many enteric pathogens, virulent *S*. Typhimurium carries a number of super oxide dismutase (SOD) genes (47, 48), and correspondingly 3% hydrogen peroxide treatment resulted in considerable gaseous oxygen production but very little toxicity (Figures 1A,B). As expected, 4% paraformaldehyde treatment and pasteurization for 1 h result in a 4–6log decrease in viable bacteria, but both methods are insufficient for the 10–11log decrease that we are aiming for (Figures 1A,B). 0.4% peracetic acid, presumably due to the combination of low pH and very strong oxidizing activity, cannot be fully inactivated by SOD enzymes. This treatment results in very minimal gas production and inactivates 10^10^ CFU *S*. Typhimurium to sterility, as determined by plating or enrichment culture (Figures 1A,B). Very little lysis of *S*. Typhimurium was observed with any of the procedures tested (Figure 1C). Light microscopy revealed largely intact bacterial bodies after inactivation (Figure 1D). Sytox-green uptake, determined by microscopy (Figure 1D) or flow cytometry (data not shown), revealed uniform loss of membrane integrity in the peracetic acid-inactivated bacteria.

To demonstrate that the efficiency of peracetic acid treatment was really sufficient for our needs, we used streptomycin pre-treated mice carrying a low-complexity microbiota, which are extremely sensitive to oral infection with streptomycin-resistant *Salmonella* (49). Twenty four hours after streptomycin treatment, these mice received just 10 or 100 CFU of live wild-type *S*. Typhimurium [strain SB300 (44)] or 5 × 10^10^ particles of peracetic acid-inactivated wild-type (SB300) or avirulent [Δ*sseD* Δ*invG* (50)] *S*. Typhimurium. Three days post-gavage, all mice receiving live *S*. Typhimurium, but 0/10 mice gavaged with peracetic acid-inactivated bacteria had full-blown cecal inflammation (Figures 1E,F) and high counts of *S*. Typhimurium in the intestinal content and mLN (Figures 1G,H). Thus, incubation in 0.4% peracetic acid is sufficient to kill >99.9999999% of all bacteria, preventing any pathological sequelae even when fully virulent wild-type *S*. Typhimurium was used as the vaccination strain.

**Figure 2:**
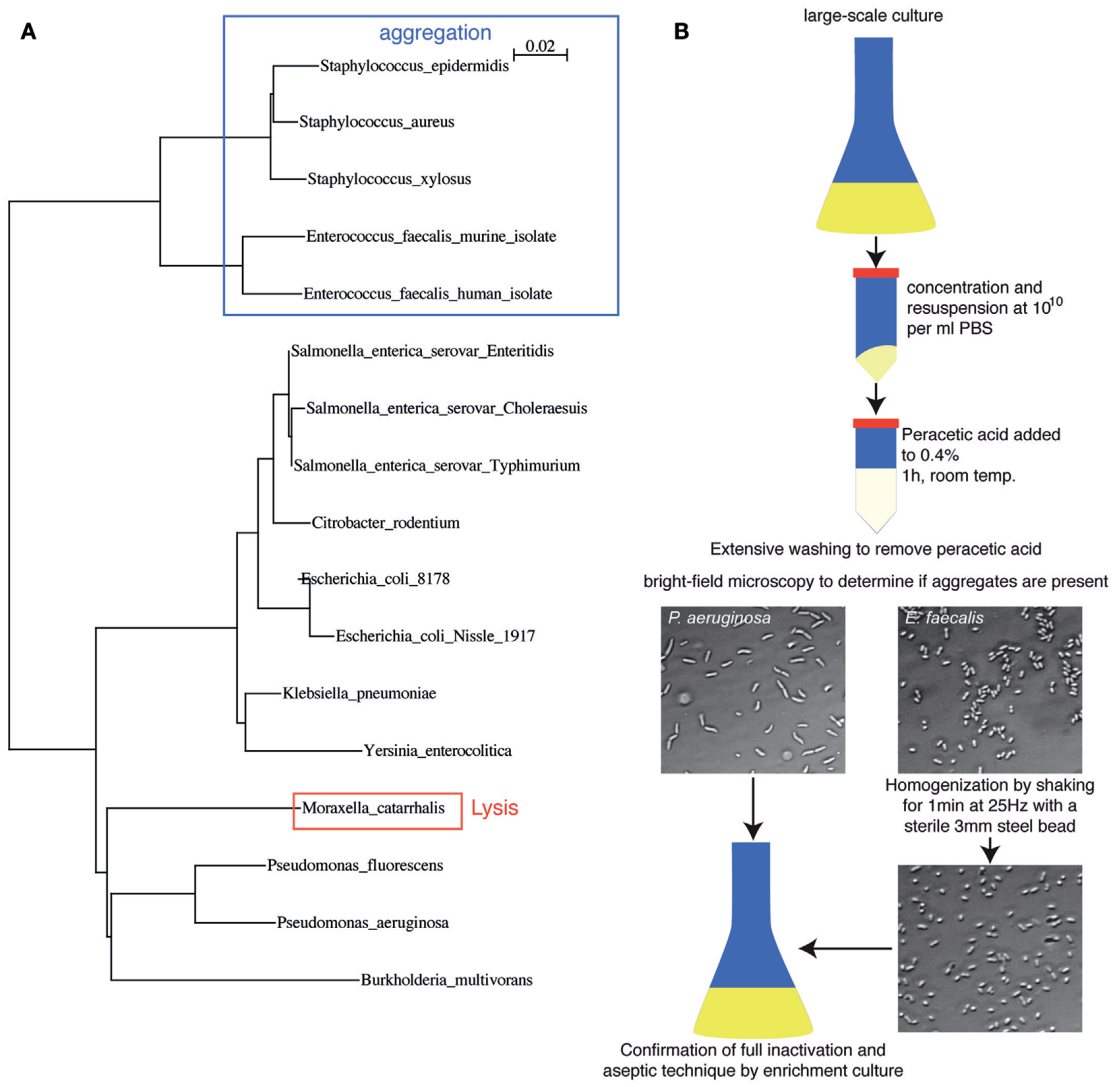
**Peracetic acid can be used to inactivate a broad range of bacterial species relevant to intestinal immunology research**. **(A)** Dendrogram based on 16S rDNA sequence differences of the species tested so far, highlighting potential pitfalls. Legend refers to the distance score as calculated by ClustalX neighbor-joining multiple alignment. **(B)** Schematic diagram of oral vaccine production, including homogenization to disrupt bacterial clumps produced during inactivation, and expected brightfield images.

In order to demonstrate that this process is applicable across a range of bacterial species, we aerobically cultured 17 different pathogenic and commensal bacterial species from the *Proteobacteria* and *Firmicutes* phyla and examined their inactivation by peracetic acid (Figure 2A). Full inactivation of dense bacterial suspensions was observed for all species tested. Spontaneous lysis in 0.4% peracetic acid was observed in only one species tested (*Moraxella catarrhalis*). Staphylococci and Enterococci tended to aggregate during inactivation, which is likely to inhibit sampling by the mucosal immune system. However, these aggregates could be easily disrupted by physical force, for example, vigorous shaking with a large steel bead (Figure 2B). The basic protocol we suggest (see Materials and Methods) is, therefore, a 1 h treatment of bacteria suspended at 10^10^ particles per ml in Dulbecco’s PBS (D-PBS) with 0.4% peracetic acid. After extensive washing with sterile D-PBS, a small aliquot of bacteria should be examined by standard light microscopy techniques to determine the extent of aggregation (or lysis if this is not macroscopically obvious). If aggregates are present, the vaccine can be homogenized by shaking at 25 Hz in the presence of a large sterile steel bead. The final vaccine preparation is resuspended with at least 10^10^ particles per 100 μl and a full 100 μl is taken into 200 ml appropriate sterile media for overnight culture to determine absolute sterility (Schematic diagram, Figure 2B). During this time, produced vaccine can be safely stored at 4°C. This is a broadly applicable, highly efficient bacterial inactivation technique that permits working with highly concentrated bacterial slurries in situations where administration of very few live bacteria would be confounding. A useful side-observation from our work is that in the case of vaccine made from *S*. Typhimurium, the inactivated bacteria could be stored as a dense slurry in PBS at 4°C for at least 3 weeks.

**Figure 3:**
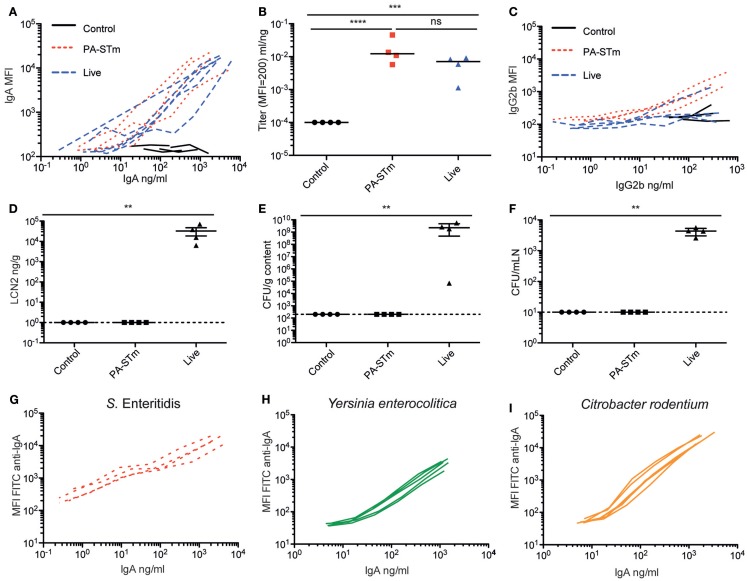
**Oral PA-STm is a strong inducer of specific intestinal IgA in the absence of pathology**. C57BL/6 SOPF mice were either pre-treated with 1.0 g/kg streptomycin and infected orally with 5 × 10^7^ CFU of the oral vaccination *S*. Typhimurium strain M556 (SB300 Δ*sseD*) (“live”) or were gavaged once a week with 10^10^ particles of peracetic acid-killed *S*. Typhimurium (“PA-STm”) over 3 weeks. **(A)** Intestinal lavage IgA titer curves and **(B)** intestinal lavage IgA titers, as calculated in Figure S3 in Supplementary Material (Kruskal–Wallis test on log-normalized values, *P* < 0.0001, Pairwise comparisons calculated by Dunn’s post-tests). **(C)** Serum IgG2b titer curves at day 21 after the first vaccination/infection, as determined by bacterial flow cytometry. **(D)** Lipocalin 2 in feces at day 21 after the first vaccination/infection (Kruskal–Wallis test, *P* = 0.0054 with Dunn’s post-test). **(E,F)** CFU of live *S*. Typhimurium recovered from the cecal content and mesenteric lymph nodes at the same time-point. One representative experiment of two shown. **(G–I)** Specific IgA induced by vaccination with peracetic acid-killed vaccines generated from with *S*. Enteritidis*, Yersinia enterocolitica*, and *Citrobacter rodentium*. Titers were determined by flow cytometry and ELISA. *N* = 5 mice per vaccine tested.

We next compared the induction of specific intestinal IgA by orally delivered peracetic acid-inactivated bacteria and a standard live-attenuated *Salmonella* vaccine strain *sseD::aphT* (18, 50). When C57BL/6 mice were pre-treated with high-dose streptomycin and then infected with *S*. Typhimurium *sseD::aphT* (50), they developed a self-limiting intestinal inflammation and robust *S*. Typhimurium-specific intestinal immunity. In parallel, we constructed a peracetic acid-inactivated vaccine from a fully avirulent *S*. Typhimurium mutant, henceforth, referred to as PA-STm (*ΔsseD ΔinvG* – M2702, used to minimize operator risk during vaccine preparation). 10^10^ particles of PA-STm were gavaged once per week for 3 weeks without antibiotic pre-treatment. On day 21 after the vaccination start, PA-STm-treated mice developed a *S*. Typhimurium-specific intestinal IgA titer that is equivalent to that observed at 3 weeks post-infection with the live attenuated strain *S*. Typhimurium M556, as determined by bacterial flow cytometry (45) (Figures 3A,B; Figure S1 in Supplementary Material). Serum IgA and IgM responses were also equivalent between the two treatments (Figure S2 in Supplementary Material). Serum IgG2b (Figure 3C) and IgG1 (Figure S2 in Supplementary Material) responses induced by PA-STm are low, but higher than those observed at the same time-point during infection with the live-attenuated vaccine. Mice receiving PA-STm displayed zero detectable intestinal inflammation as determined by histopathology scoring (data not shown), or by quantification of fecal Lipocalin 2 (Figure 3D) (51). Furthermore, no live *S*. Typhimurium was recovered from the cecum content or from draining lymphoid tissues of mice receiving high-dose peracetic acid-inactivated bacteria, whereas this is observed in 100% of mice vaccinated with live-attenuated strains at this time-point (Figures 3E,F). To demonstrate that this robust induction of specific IgA is not restricted to *Salmonella* Typhimurium, we tested peracetic acid-inactivated oral vaccines from a range of *Enterobacteriaceae* species *in vivo*. We could demonstrate robust induction of specific IgA against two other non-typhoidal *Salmonella* serovars (Enteritidis – Figure 3G, Choleraesuis – data not shown), as well as the more distantly related species *Yersinia enterocolitica* and *Citrobacter rodentium* (Figures 3H,I).

**Figure 4:**
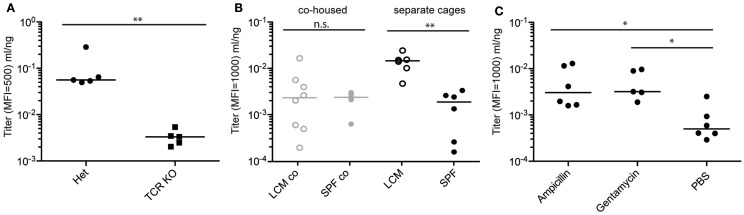
**Specific IgA induction by PA-STm is dependent on T cells and the microbiota**. **(A)** TCRβδ^−/−^ and matched heterozygote controls were vaccinated three times over 3 weeks with PA-STm. On day 21, after the first vaccination, all mice were euthanized, and IgA in the intestinal lavage analyzed by bacterial flow cytometry for *Salmonella* specific IgA, and ELISA for total IgA concentrations. Pooled data from two independent experiments. Mann–Whitney *U* test *P* = 0.0079. **(B)** Female LCM and SPF mice were either co-housed for 3 weeks, or were housed separately under identical conditions. Subsequently, all mice were gavaged three times over 3 weeks with PA-STm. Antibody titers were determined as above on day 21 after the initial vaccination. Pooled data from three independent experiments. Two-way ANOVA *P* (hygiene effect) = 0.0142, *P* (Interaction between housing and hygiene) = 0.0104. **(C)** C57BL/6 SPF mice were pre-treated orally with vehicle only (PBS) or high-dose ampicillin (0.8 g/kg) or gentamycin (1 g/kg) 24 h prior to each PA-STm dose. Three rounds of pre-treatment and vaccination were carried out over 3 weeks. On day 21, antibody titers were determined as in **(A)**. Pooled data from two independent experiments. Kruskal–Wallis *P* = 0.0140 with Dunn’s post-test.

We next tested the host requirements for induction of specific IgA by peracetic acid-inactivated *Salmonella*. As expected for a high-affinity antibody responses (52), induction of high-titer IgA by PA-STm was abroaged in TCRβ^−/−^ TCRδ^−/−^ mice (lacking the entire T cell compartment), even when lower total IgA production was taken into account, indicating that the inactivated vaccine is also capable of eliciting a T follicular helper response facilitating specific IgA production (Figure 4A). Importantly, we observed a strong quantitative effect of different microbiota compositions on *S*. Typhimurium-specific IgA titers, which roughly correlated with hygiene status (Figure 4B). Mice with a very limited microbiota “LCM – low complexity microbiota” (23) (LCM – separate cages, Figure 4B), or mice recently re-derived into an ultra-clean SOPF foster colony (Figure 3A), produce very high IgA titers with little mouse-to-mouse variation. By contrast, mice bred in a SPF colony that harbors a more diverse microbiota, including low levels of protozoa (SPF – separate cages, Figure 4B), produced lower and more variable IgA titers specific for *S*. Typhimurium when vaccinated in parallel. Cohousing of LCM mice with animals with a diverse SPF microbiota for 3 weeks prior to commencement of vaccination significantly decreased the titer of *S*. Typhimurium-specific IgA produced (co-housed, Figure 4B). Correspondingly, pre-treatment of SPF animals with ampicillin or gentamicin 24 h prior to each vaccination increased final antibody titers (Figure 4C). However, as minimal disruption of the microbiota community was a major aim in developing our inactivated vaccination protocol, antibiotic-mediated augmentation of the vaccination response was not further employed. While it is beyond the scope of the current investigation to determine the exact nature of the “transferrable” microbiota that mediates this effect, it will clearly be critical to control for microbiota composition in all studies employing inactivated oral vaccines.

**Figure 5:**
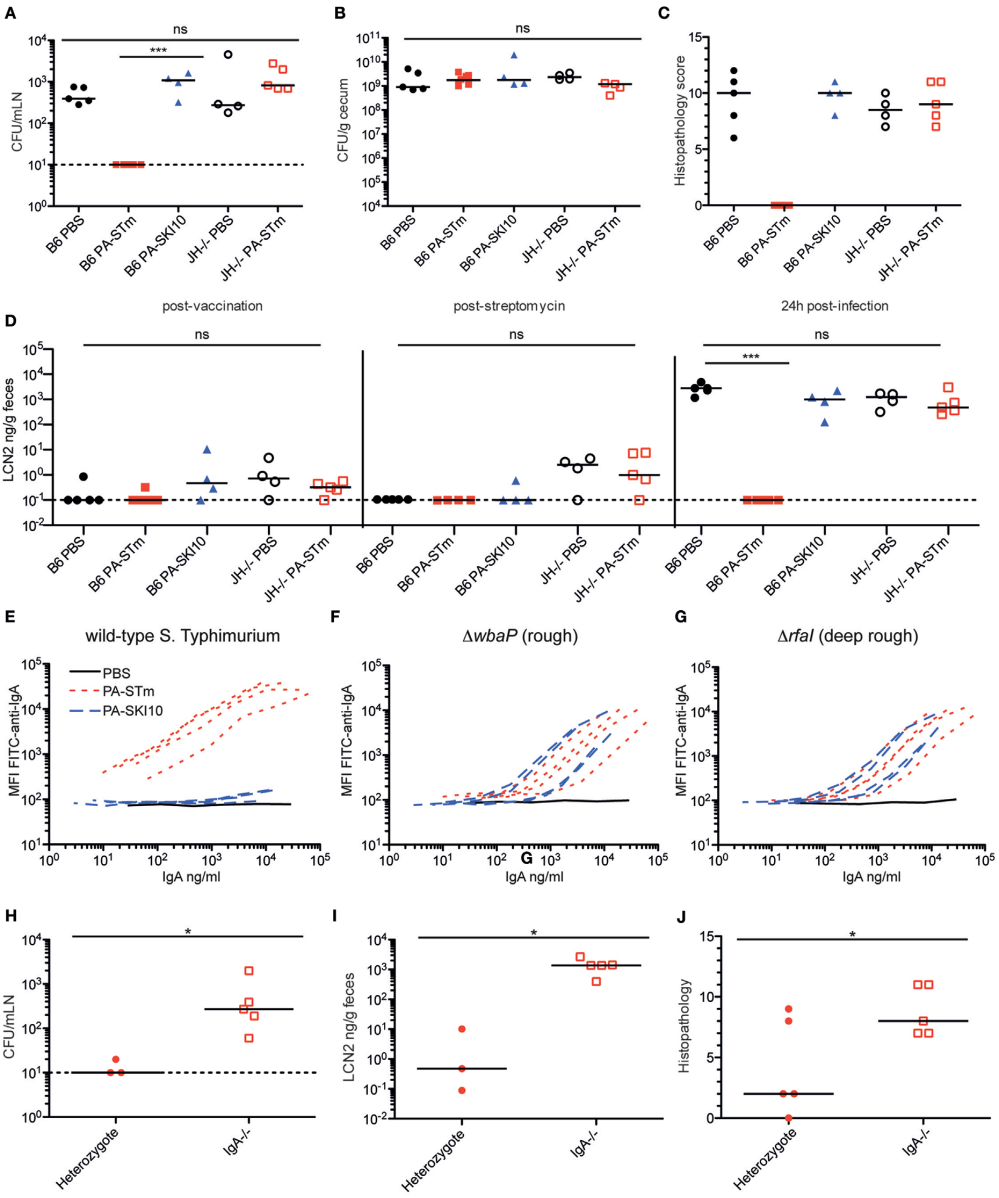
**PA-STm provides protection from non-typhoidal *Salmonellosis* in an O-antigen and antibody-dependent manner**. **(A)** C57BL/6 SOPF or JH^−/−^ mice recently rederived into an SOPF colony were vaccinated once per week for 3 weeks with the indicated vaccine (PA-STm: killed O-antigen-sufficient vaccine, PA-SKI10: killed O-antigen-deficient “rough” strain). On day 21, all mice were pre-treated with 1.0 g/kg streptomycin p.o. 24 h later, all mice received 10 CFU of wild-type *S*. Typhimurium SB300 p.o. Mice were euthanized 24-h post-infection. **(A,B)** Live *S*. Typhimurium CFU in the mesenteric lymph nodes (Kruskal–Wallis *P* = 0.0009, with Dunn’s post-tests) and cecal content. **(C)** Histopathology of the cecum at 24-h post-infection. (Kruskal–Wallis test *P* = 0.0325) **(D)** Fecal Lipocalin 2 on day 21 post-vaccination, 24 h post streptomycin treatment and 24 h post-challenge (24 h post-challenge, Kruskal–Wallis *P* = 0.0006, with Dunn’s post-tests). **(E–G)** Intestinal IgA titer specific for the surface of wild-type *S*. Typhimurium, rough *S*. Typhimurium (Δ*wbaP*) and deep-rough *S*. Typhimurium (ΔrfaI), as determined by bacterial surface-specific bacterial flow cytometry. **(H–J)** IgA^±^ and IgA^−/−^ SOPF littermate mice were vaccinated three times over 3 weeks with PA-STm. All mice were streptomycin pre-treated, followed by infection with 10^5^ CFU wild-type *S*. Typhimurium. All parameters were assessed 24-h post-infection. **(H)** CFU of live *S*. Typhimurium in the mesenteric lymph nodes (Mann–Whitney *U P* = 0.0358). **(I,J)** Intestinal pathology as determined by fecal Lipocalin 2 levels (Mann–Whitney *U P* = 0.0357) and histopathology (Mann–Whitney *U P* = 0.0336).

We next carried out proof-of-principle experiments to determine whether intestinal responses to peracetic acid-inactivated vaccines are functional. To this end, we made use of the murine model of invasive non-typhoidal *Salmonellosis* (36). In this model, SOPF mice received high-dose oral streptomycin 24 h prior to infection to reduce the density of the microbiota in the large intestinal lumen and generate a permissive niche for *S*. Typhimurium growth. 5 × 10^5^ *S*. Typhimurium were given orally and grew to a density of 10^9^–10^10^ CFU per gram intestinal content, filling this niche by day 1 post-infection. Once bacteria reach a sufficiently high density, virulence factor expression is triggered and *Salmonella* can invade into epithelial cells and penetrate to draining lymph nodes and systemic sites (36). This is a particularly challenging model for vaccine-mediated protection as the lethal infectious dose in C57BL/6 mice is <10 CFU. Previous work in this model using live-attenuated vaccination had demonstrated that O-antigen-specific IgA is a necessary component of any observed protection (18). Therefore, we additionally tested the effect of PA-STm in antibody-deficient animals [J_H_^−/−^ (38)] and the effect of a vaccine produced from a “rough” (i.e., O-antigen-deficient) *S*. Typhimurium strain SKI10 (*wbaP:aphT*) (53). PA-STm vaccinated wild-type mice were completely protected from an oral challenge of 5 × 10^5^ *S*. Typhimurium in the streptomycin pre-treatment model at 24 h post-infection, with no detectable live *S*. Typhimurium recovered from the mLN, despite high levels of cecal colonization (Figures 5A,B). Additionally, no cecal pathology was observed in wild-type mice vaccinated with PA-STm as determined by histopathology (Figure 5C) or fecal Lipocalin 2 (Figure 5D). By contrast, mice vaccinated with a peracetic acid-inactivated strain lacking the O-antigen (PA-SKI10) showed no protection either at the level of cecal pathology or tissue bacterial loads (Figures 5A–D), and no IgA binding to wild-type *Salmonella* (Figure 5E). This was not due to reduced antigenicity of this vaccine as IgA induce by PA-SKI10 was as good as that induced by PA-STm in binding to surface structures of O-sidechain-deficient *S*. Typhimurium mutants (rough or deep-rough strains, Figures 5F,G). Rather, the presence of O-sidechains masks most other relevant antigens on the surface of live bacteria. As would be predicted from previous work (18), antibodies were an essential component of the protective response, as PA-STm vaccinated J_H_^−/−^ mice, which lack all mature B cells (38), show no measurable protection from infection (Figures 5A–E). Furthermore, vaccinated IgA-deficient mice display greatly reduced protection from infection (Figures 5H–J), when compared to IgA heterozygote littermates. Therefore, the main protective immune response induced by PA-STm was O-antigen-specific IgA.

**Figure 6:**
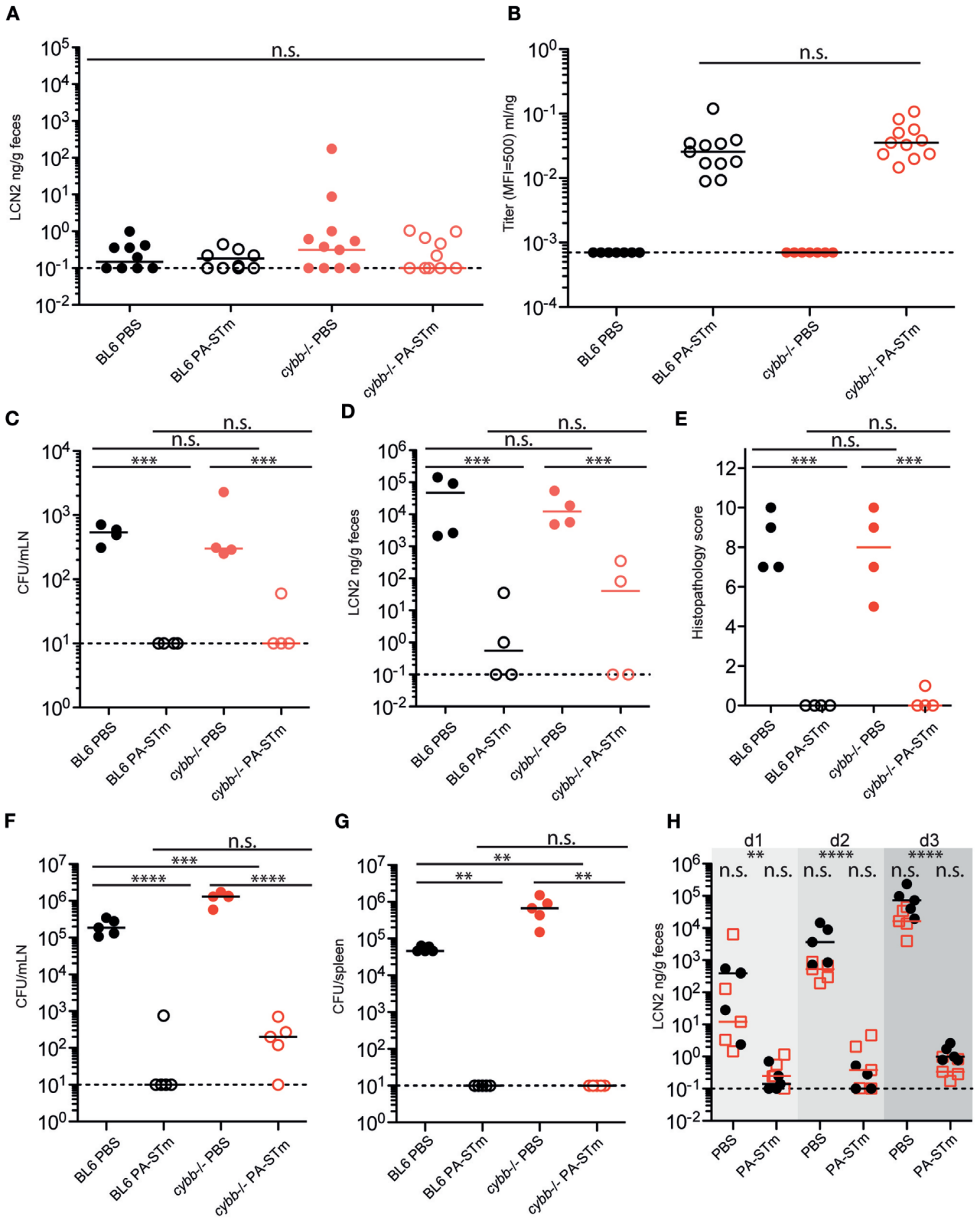
**Oral PA-STm is safe for vaccination in *cybb*-deficient mice and provides dose-dependent protection from tissue invasion and pathology up to at least 80-h post-infection in the non-typhoidal *Salmonellosis* model**. C57BL/6 and *cybb*^−/−^ mice recently re-derived into an identical SOPF foster colony were vaccinated three times over 3 weeks with PA-STm or vehicle alone (PBS). **(A,B)** On day 21 after the first vaccination, cecal pathology was determined by fecal Lipocalin 2 ELISA [two-way ANOVA *P* (genotype) = 0.3583, *P* (vaccination) = 0.3500, *P* (Interaction) = 0.3515] **(A)** and intestinal IgA titers were determined by bacterial flow cytometry (Mann–Whitney *U* test on vaccinated samples only, *P* = 0.1679) **(B)**. **(C–E)** Mice vaccinated as in A and B were pre-treated with 1 g/kg streptomycin on day 21 after the first vaccination and subsequently infected with 10^5^ wild-type *S*. Typhimurium. **(C)** Live *S*. Typhimurium CFU recovered from the mesenteric lymph nodes at 24-h post-infection. **(D,E)**. Intestinal inflammation as determined by fecal Lipocalin 2 **(D)** and histopathology scoring of cecum tissue **(E)**. **(C–E)** were analyzed by (two-way ANOVA, ***P (vaccination) <0.001) **(F–H)**. Mice vaccinated as in **(A,B)** were pretreated with 1 g/kg streptomycin on day 21 after the first vaccination and subsequently infected with 50 CFU wild-type *S*. Typhimurium. **(F,G)** Live *S*. Typhimurium CFU recovered from the mesenteric lymph nodes **(F)** [two-way ANOVA. *P* (genotype) = <0.0001, *P* (vaccination) <0.0001, *P* (interaction) = <0.0001] and spleen **(G)** (two-way ANOVA. *P* (genotype) = 0.0098, *P* (vaccination) = 0.0038, *P* (interaction) = 0.0098) at 80-h post-infection. **(H)** Intestinal inflammation as determined by fecal Lipocalin 2 up to 80 h (day 3) post-infection (two-way repeat-measures ANOVA with Bonferroni post-tests on log-normalized data. **P < 0.01, ****P < 0.0001).

The sterile nature of PA-STm immediately suggested its potential to investigate mucosal immunity in situations of severe innate immune deficiency. Both human patients and mice carrying mutations in the phagocyte NADPH oxidase component gp91phox (*cybb*) are extremely susceptible to invasive *Salmonellosis* and also develop overt pathology when infected with live-attenuated oral vaccination strains (37, 54). In contrast to live-attenuated *Salmonella*, PA-STm was extremely well tolerated in *cybb*-deficient animals with no statistical difference in inflammatory scores or intestinal inflammatory markers between mock-treated and vaccinated mice (Figure 6A, data not shown).

Additionally, the *cybb* pathway is not required for successful responses to the vaccine, as titers of intestinal IgA (Figure 6B) are similar to those of matched wild-type mice. Critically, this adaptive immune response is equally protective in *cybb*-deficient and wild-type mice up to 24 h post-infection with a large inoculum (5 × 10^5^ CFU, Figures 6C–E), and up to at least 80 h post-infection with a small inoculum (50 CFU, Figures 6F–H).

## Discussion

Based on our previous observations with apathogenic species, we devised and tested a highly simplified strategy to generate high dose inactivated oral vaccines, capable of inducing robust specific intestinal IgA responses. The strong oxidizing agent peracetic acid has long been used as a decontaminant in the husbandry of axenic animals (55) and in the food industry (56, 57). Here, we demonstrate that the bacterial killing efficiency of 0.4% peracetic acid is considerably higher than those of standard vaccine inactivation protocols (4% paraformaldehyde, pasteurization, or 3% hydrogen peroxide), and can be used on a wide range of bacterial species. This permits the oral application of very high numbers of vaccine particles with close-to-zero risk of inoculating live bacteria (Figures 1 and 2). When sufficient numbers of inactivated bacteria are delivered orally, we can induce a robust T-cell-dependent mucosal IgA response against a range of *Enterobacteriaceae* (Figures 3 and 4). This occurs in the complete absence of intestinal pathology, in the complete absence of live bacteria and in the absence of exogenous mucosal adjuvants (Figure 3), even in situations of severe innate immune deficiency (Figure 6). The immune responses induced can protect from oral infection with virulent *S*. Typhimurium in the mouse model of non-typhoidal *Salmonellosis* (Figures 5 and 6). This is the first demonstration of phagocyte oxidative burst-independent protection by high-titer IgA in this infectious model. Our observation somewhat goes against a prevailing dogma in mouse vaccination that sterile material delivered orally induces either tolerance or is simply ignored by the immune system (12, 13, 58). While both phenomena can be easily observed, our clear demonstration of immunity highlights the quantitative nature of mucosal immune system stimulation.

The beauty of this strategy is that it permits researchers using well-established animal models of host–microbiota interactions or host–pathogen–(microbiota) interactions to generate high-titer IgA responses in an otherwise largely unperturbed host. Highly controlled infections or colonizations can then be carried out to determine the mechanisms by which IgA alters microbial physiology *in vivo* (for example, bacterial virulence, induction of inflammatory signaling, within-host population dynamics, and within-host evolution). When combined with very rigorous aseptic technique, this methodology should be safe enough to apply in germ-free and gnotobiotic animals without contamination (55). Extra caution would need to be applied with species capable of forming spores, which may be highly resistant (59) and hard to detect by *in vitro* enrichment culture. Another benefit of the technique is that the antigenic composition of the vaccine is determined by *in vitro* growth and the bacterial phenotype becomes locked at the time of inactivation. Therefore, there is the possibility to grow the vaccination strain under selection to maintain phase-locked states, or to overexpress factors associated with a high metabolic cost. This is almost impossible to achieve with live vaccines due to rapid out-selection of the fittest strains *in vivo* (60). Of note, the more pessimistic reader may spot that this is also a potential disadvantage, as bacteria grown in rich media *in vitro* may be antigenically distinct from those *in vivo* and modification of the culture conditions may be important to generate appropriate responses.

We envisage this technique as being immediately useful to the field of host–microbial interaction in experimental animal models. Based on our previous work, we expect the induced IgA responses to wane quite rapidly after the final vaccine dose (13) and, therefore, the usefulness in human and veterinary medicine may be limited. However, it should be noted that two licensed human oral vaccines against *V. cholera* and one vaccine in late-stage clinical trials against ETEC (31) work on the basis of high numbers of bacteria killed by formalin or heat-treatment (30). All of these vaccines include an autologous toxin (or subunit thereof) which is a known mucosal adjuvant (61). It remains unclear whether the absence of long-term-mucosal memory observed with non-adjuvanted mucosal vaccines in the murine system is due to physiological differences, or the absence of adjuvant. The greatly increased efficiency of peracetic acid-mediated inactivation, as compared to paraformaldehyde and heat-treatment, suggests that this process could be used to increase the efficiency of human oral vaccines adjuvented with cholera toxin B or heat-labile toxin B subunit. It will additionally be interesting to test these adjuvanted vaccines to look for qualitative and quantitative differences in activation of mucosal immunity in animal models. Furthermore, there are a number of clinical situations where highly susceptible patients could benefit from a short-term extremely safe boost in mucosal immunity against diverse bacterial strains, such as prior to myeloablation, in primary innate immune deficiencies, during TNF-blockade, or prior to fecal transplantation. In these situations, the ability to easily produce oral vaccines from a broad range of bacterial species may be of clinical interest.

Currently, we have not tested whether this technique is also suitable for inactivation of strictly anaerobic bacterial species. A further limitation is that we have not investigated the nature of T cell responses induced by peracetic acid-inactivated oral vaccines. Additionally, we observe an effect of microbiota composition on the efficiency of specific IgA induction by peracetic acid-inactivated vaccines, which suggests that there will be lab-to-lab variation in absolute efficacy. Despite these caveats, we feel this technique finally makes it possible to cleanly study the effects of the antibody-mediated intestinal immunity on host-microbial interactions.

## Author Contributions

KM desgined and carried out experiments, reviewed the manuscript, and analyzed data; SW carried out experiments; AT assisted with experiments; MD blind-scored histopathology; SH collaborated on developing the initial method; and ES developed the methods shown, designed and carried out experiments, analyzed data, and wrote the manuscript.

## Conflict of Interest Statement

The authors declare that the research was conducted in the absence of any commercial or financial relationships that could be construed as a potential conflict of interest.
