# Supplementary material for: Peracetic Acid Treatment Generates Potent Inactivated Oral Vaccines from a Broad Range of Culturable Bacterial Species
Source: Front Immunol. 2016 Feb 11;7:34. doi: 10.3389/fimmu.2016.00034 (PMC4749699; doi:10.3389/fimmu.2016.00034)
Supplement: Supplementary file 1 [file image_1.pdf]

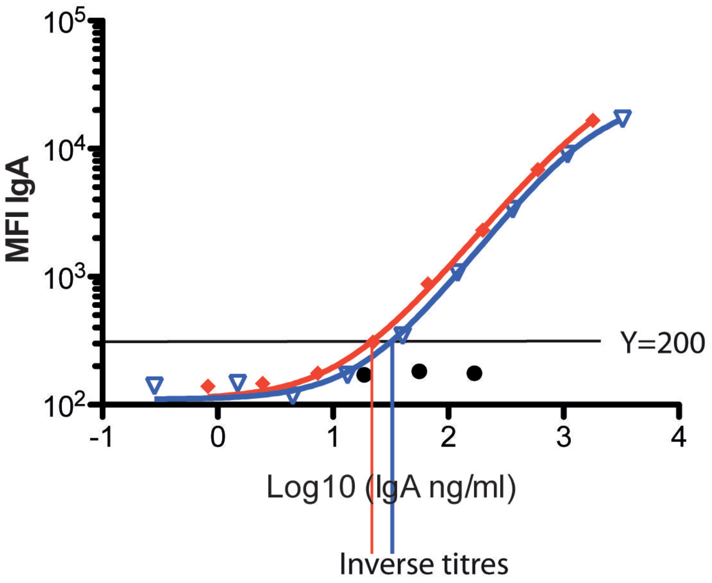

Supplementary Figure 1: Calculation of antibody titers from bacterial flow cytometry data. Live bacterial targets were stained with dilution series of serum or intestinal lavages. Total antibody concentrations in serum and lavages was determined by sandwich ELISA. Median fluorescence intensity (MFI) was calculated for each flow cytometry sample and plotted against the total antibody concentration in each dilution series step (IgA in this example). 4-parameter logistic curves were fitted to each data series using Graphpad Prism by least-squares nonlinear regression. The concentration of total antibody required to achieve a given MFI (in the example =200) was calculated by re-arrangement of the fitted 4-parameter logistic equation for each sample. As this value is low where a strong antibody response is present, the inverse of this value was plotted. Thus, titers are calculted as the inverse total antibody concentration required to achieve a given (MFI). The y-axis value chosen as “above background” necessarily varies between experiments due to the flow cytometer settings, but is constant within any one analysis.
