# Supplementary material for: Peracetic Acid Treatment Generates Potent Inactivated Oral Vaccines from a Broad Range of Culturable Bacterial Species
Source: Front Immunol. 2016 Feb 11;7:34. doi: 10.3389/fimmu.2016.00034 (PMC4749699; doi:10.3389/fimmu.2016.00034)
Supplement: Supplementary file 2 [file image_2.pdf]

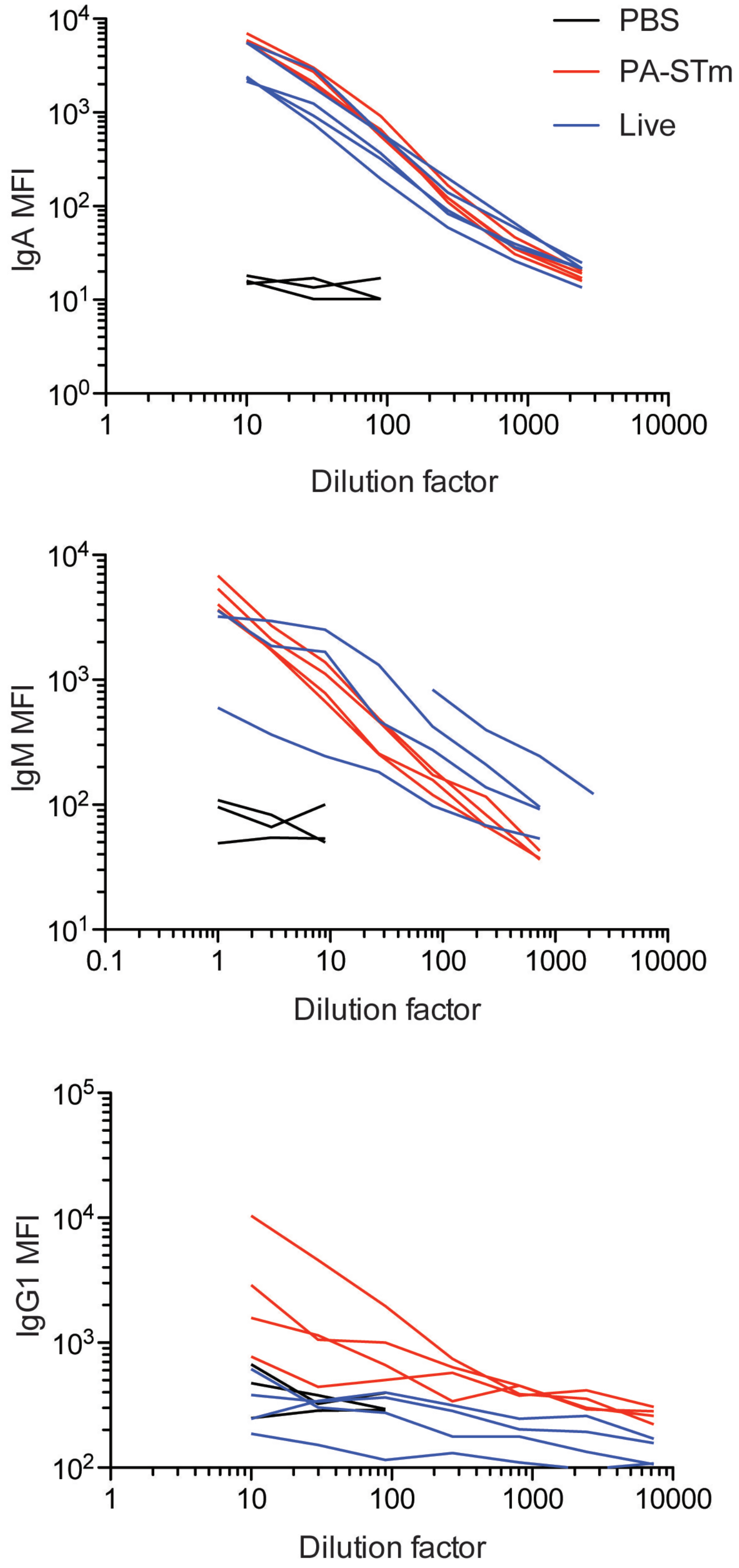

Supplementary Figure 2. Serum antibody titration curves against *S.* Typhimurium corresponding to Figure 3A-C. C57BL/6 SOPF mice were either pre-treated with 1.0g/kg streptomycin and infected orally with $5x10^7$ CFU of the oral vaccination *S.* Typhimurium strain M556 (SB300 ΔsseD) ("Live") or were gavaged once a week with 10^{10} particles of peracetic acid-killed S. Typhimurium ("PA-STm") or vehicle only ("PBS") over three weeks. Serum was collected on d21 after the first gavage/infection (d7 after the final PA-STm gavage) and analysed by bacterial flow cytometry.
